# Oral squamous cell carcinoma grading classification using deep transformer encoder assisted dilated convolution with global attention

**DOI:** 10.3389/frai.2025.1575427

**Published:** 2025-10-17

**Authors:** Singaraju Ramya, R. I. Minu

**Affiliations:** Department of Computing Technologies, School of Computing, SRM Institute of Science and Technology, Chennai, India

**Keywords:** GAN model, adaptive bilateral filter, U-net model, dilated convolutional, Grey lag goose optimization algorithm and global attention

## Abstract

In recent years, Oral Squamous Cell Carcinoma (OSCC) has been a common tumor in the orofacial region, affecting areas such as the teeth, jaw, and temporomandibular joint. OSCC is classified into three grades: “well-differentiated, moderately differentiated, and poorly differentiated,” with a high morbidity and mortality rate among patients. Several existing methods, such as AlexNet, CNN, U-Net, and V-Net, have been used for OSCC classification. However, these methods face limitations, including low ACC, poor comparability, insufficient data collection, and prolonged training times. To address these limitations, we introduce a novel Deep Transformer Encoder-Assisted Dilated Convolution with Global Attention (DeTr-DiGAtt) model for OSCC classification. To enhance the dataset and mitigate over-fitting, a GAN model is employed for data augmentation. Additionally, an Adaptive Bilateral Filter (Ad-BF) is used to improve image quality and remove undesirable noise. For accurate identification of the affected region, an Improved Multi-Encoder Residual Squeeze U-Net (Imp-MuRs-Unet) model is utilized for segmentation. The DeTr-DiGAtt model is then applied to classify different OSCC grading levels. Furthermore, an Adaptive Grey Lag Goose Optimization Algorithm (Ad-GreLop) is used for hyperparameter tuning. The proposed method achieves an accuracy (ACC) of 98.59%, a Dice score of 97.97%, and an Intersection over Union (IoU) of 98.08%.

## 1 Introduction

Oral Squamous Cell Carcinoma (OSCC) is one of the most prevalent malignancies affecting the oral cavity and remains a major cause of morbidity and mortality worldwide. Despite advances in diagnostic tools, the prognosis of OSCC patients continues to depend largely on the stage and grade of the disease at the time of detection (Chu et al., 2021). Early and accurate identification of tumor grade is therefore crucial for guiding treatment strategies and improving survival outcomes.

Traditional histopathological diagnosis, while effective, relies heavily on the expertise of pathologists and is prone to inter-observer variability, leading to inconsistencies in classification (Sukegawa et al., 2023). With the growing volume of biopsy samples, manual examination has become increasingly challenging, often resulting in delays and diagnostic inaccuracies (Yoshizawa et al., 2022). These challenges highlight the need for automated, reliable systems that can assist clinicians in achieving more consistent and efficient diagnostic outcomes.

In recent years, deep learning (DL) has emerged as a powerful tool in medical image analysis, enabling significant progress in cancer detection, segmentation, and classification. Several studies have explored CNN-based and transformer-based approaches for OSCC and related cancers. For instance, Wako et al. (2022) applied transfer learning (TL) with CNNs for margin classification of squamous cell carcinoma, reporting strong performance but noting reduced accuracy in the absence of hybrid models. Alanazi et al. (2022) introduced an intelligent DL-enabled OSCC detection framework combining NasNet features with a deep belief network, achieving promising accuracy but limited generalization to unseen data. Similarly, Albalawi et al. (2024) employed EfficientNet B3 for OSCC classification on histopathological images, though performance was constrained by dataset size. Peng et al. (2024) investigated various TL models such as Inception v4, ShuffleNet V2, and ResNet 50 for Oral Epithelial Dysplasia (OED) grading, but accuracy improvements remained modest. Das et al. (2024) developed an ensemble model combining CNN classifiers, achieving 97.88% accuracy, though their work was restricted to binary classification. Beyond histopathology, Flügge et al. (2023) utilized transformers for OSCC detection in clinical photographs, while Li et al. (2024) combined MRI-based transformers with radiomics for early- and late-stage OSCC detection.

Collectively, these studies demonstrate the potential of deep learning for OSCC analysis. However, challenges such as limited datasets, over fitting, computational inefficiency (Deif et al., 2022), absence of hybrid approaches, and poor real-time applicability persist. Moreover, most existing works focus on binary classification rather than multi-class grading (Ananthakrishnan et al., 2023), which is critical for clinical decision-making.

Motivated by these gaps, this work introduces a robust hybrid framework for OSCC classification and grading from histopathological images (Yang et al., 2022). The proposed approach incorporates GAN-based augmentation to address data scarcity, adaptive filtering for noise removal, an improved multi-encoder residual squeeze U-Net for segmentation, and a transformer encoder-assisted dilated convolution with global attention (DeTr-DiGAtt) for classification (Ahmad et al., 2023). Additionally, hyperparameters are optimized using the Adaptive Grey Lag Goose Optimization algorithm to enhance model efficiency. The results demonstrate that the proposed method not only achieves higher accuracy but also improves generalization, thereby reducing diagnostic subjectivity and supporting clinicians with a reliable decision-support tool (Rahman et al., 2022; Lin and Chen, 2022).

## 2 Proposed methodology

The proposed methodology is designed to achieve accurate classification and grading of Oral Squamous Cell Carcinoma (OSCC) from histopathological images through a structured multi-stage pipeline (Panigrahi et al., 2023; Das et al., 2023). First, data augmentation is performed using Generative Adversarial Networks (GANs) to address the challenge of limited and imbalanced datasets. The images are then pre-processed through resizing, normalization, and color standardization to ensure consistency across the dataset (Fatapour et al., 2023). In the next stage, segmentation is carried out using advanced deep learning-based architectures to isolate tumor regions and enhance relevant features (Haq et al., 2023; Mohan et al., 2023). Following segmentation, discriminative features are extracted using statistical and texture-based descriptors, which capture both local and global image characteristics. Finally, classification models—including hybrid deep learning networks and optimized transformers—are applied to categorize the images into normal, tumor, and graded OSCC classes (Meer et al., 2025; Dhanya et al., 2024; Kumar et al., 2024). This systematic approach enhances diagnostic accuracy while overcoming challenges related to data scarcity, intra-class variability, and complex tissue structures.

### 2.1 Data augmentation

GAN-based data augmentation (Zhang et al., 2022) can be used to reduce overfitting problems and obtain reliable improvements from the proposed method. GANs are useful for diversifying datasets by generating new models. Where C and E represent the generator and a discriminator, which is defined as following Equation 1.


(1) $$\begin{array}{l} \min_{C}\max_{E}W(C,E)=F_{y}q_{\text{data}(y)}[\log C(y)]\\ +F_{x}q_{x(x)}[\log(1-C(E(y)))] \end{array}$$

**Figure 1:**
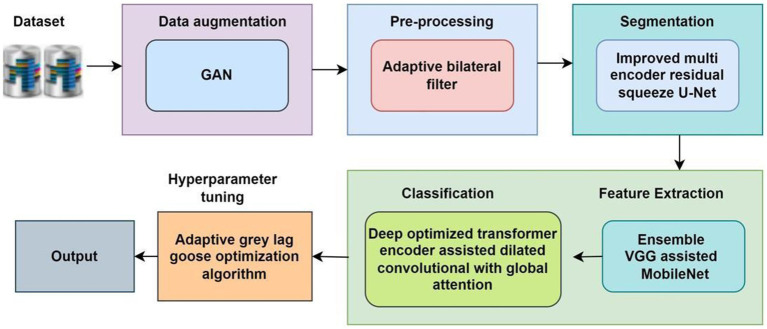
Overall flow diagram.

Where $q_{\text{data}(y)}$ represents the probability distribution of data variables y , W(C,E) represents the value function, $q_{x(x)}$ represents noise variables y _and_ and E(y) represents fake samples that are generated from the random noise y (Ghaznavi et al., 2024; Suiçmez, 2025). This has significantly augmented the overall sample count in the dataset using the GAN model (Figure 1).

### 2.2 Pre-processing

Traditional filters such as median filters and Gaussian filters have some limitations, including low-quality images and high noise. To overcome these limitations, the new method uses an adaptive bilateral filter (Bedi et al., 2024). The adaptive bilateral filter effectively removes noise from the input image defined by the following Equation 2.


(2) $$\begin{array}{l} s_{m0,p0}=\sum_{p=p_{0-M}}^{P_{o}+M}\sum_{m=m_{0--M}}^{P_{o}+M}f^{\left(-\frac{{\left(P-P_{0}\right)}^{3}+{\left(n-n_{0}\right)}^{2}}{2\sigma_{c}^{2}}\right)}\\ \times f^{\left(\frac{L(d[p,m]-d[p_{0}-m_{0}]-\xi [p_{0,m_{0}}])}{2\sigma_{s}^{2}[p_{0,m_{0}}]}\right)} \end{array}$$

Where ξ represent the range of image, $\sigma_{s}$ represent the width of the image, $m_{0},p_{0}$ represents the center of pixel window, $\sigma_{c}$ and $\sigma_{s}$ represents the standard derivation of the domain. This model has been effectively de-noised the image.

### 2.3 Segmentation using improved multi-encoder residual squeeze U-net model

**Figure 2:**
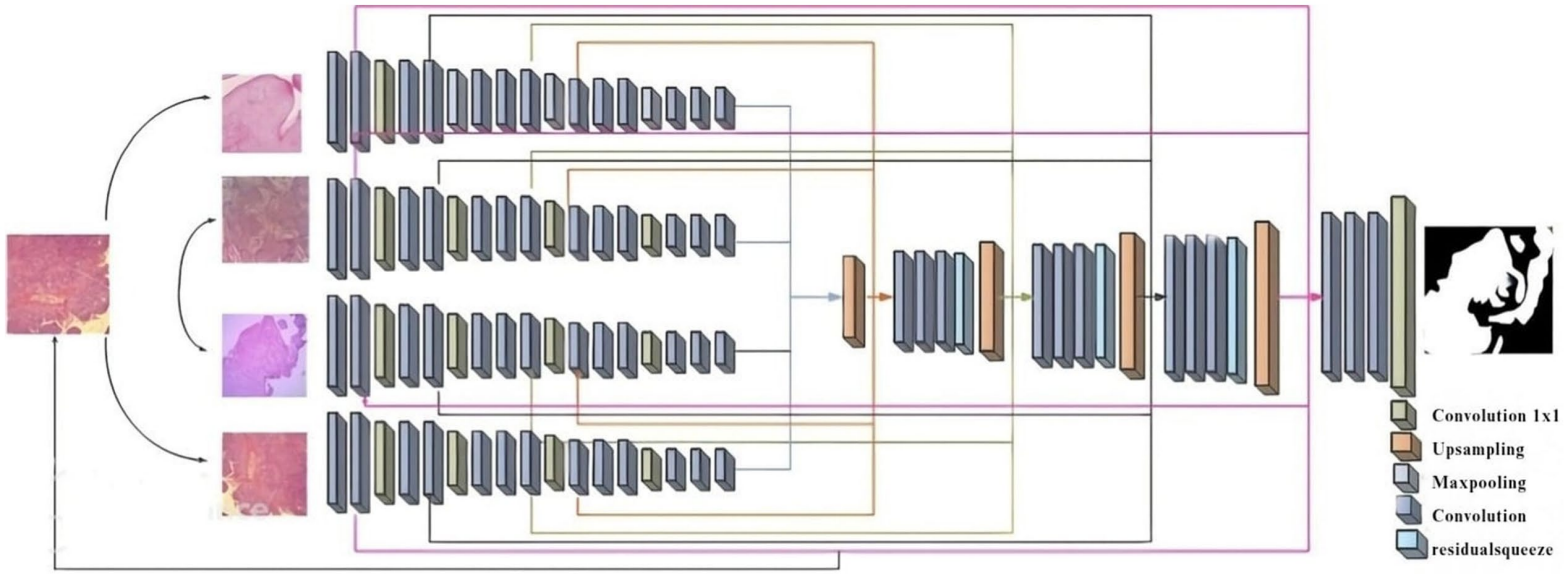
Diagram for Imp-MuRs-Unet.

The existing segmentation methods had some limitations, such as overtime duration, blurred image quality, and insufficient edges (Abuhayi et al., 2024; Islam et al., 2024). To overcome those existing limitations, introduce Figure 2 which is shown as a novel improved multi-encoder residual squeeze U-Net model.

The multi-encoder (Wang et al., 2022) input $Y\in P^{B\times L\times M}$ feature map, convolution layer is assed to each upsampling to obtain essential information of 1D channel attention map and a 2D spatial attention map. It can be define as following Equations 3, 4.


(3) $$Y\prime =N_{B}(Y)⊗Y$$

(4) $$Y^{\prime }\prime =N_{O}(Y\prime )⊗Y\prime$$

Where ⊗ represent the element-wise multiplication, $N_{O}(Y\prime )$ and $N_{B}(Y)$ represent the spatial and the channel attention map, which is calculated by following Equations 5, 6.


(5) $$N_{0}(Y)=\sigma (\mathrm{MLP}(q_{\mathrm{avg}}(Y)+q_{\max}(Y)))$$

(6) $$N_{o}\prime (Y)=\sigma \{(Y^{7\times 7}[q_{\mathrm{avg}}(Y\prime )+q_{\max}(Y\prime )])\}$$

Where $q_{\mathrm{avg}}(•)$ and $q_{\max}(•)$ represent the maximum and average pooling, MLP(•) represent the multi-layer of perceptron, $Y^{7\times 7}$ represent the operation of convolution with 7×7 filter size.

#### 2.3.1 Encoder

The input feature map $Y_{e}\in P^{B\times \frac{I}{q}\times \frac{I}{q}}$ Processed through the multi-encoder to generate a one-dimensional feature map of a predefined length. The encoder includes M layer of multi-layer perceptron (MLP) and multi-head attention (MHA). Here encode the location information by directly adding learnable level embedding to the feature map. To construct the encoder’s input sequence as Equation 7 follows:


(7) $$Y_{0}=\{q_{1}+n_{1}+n_{2}..\ldots \ldots \ldots n_{x}\}$$

The output of the MHA is then switched with the residual connection through an MLP block, which is defined as following Equation 8.


(8) $$Y_{x}=\mathrm{MHA}(\mathrm{LN}(Y_{x-1}))+\mathrm{MLP}(\mathrm{LN}(\mathrm{MHA}(\mathrm{LN}(Y_{x-1}))))$$

Where $Y_{x}$ represent the output of the $x^{\mathrm{th}}$ layer and LN represent the normalization operator.

#### 2.3.2 Decoder

For better ischemia stroke segmentation, skip-connectors are connected to decoder parallels with low-level features. The decoder system of the U-Net framework uses the Squeeze and-Excitation (SE) module (Suiçmez, 2025) to optimize the combination of “high-level and low-level characteristics.” The squeeze operation is achieved global average ensemble compress global spatial information into a single channel representation, which is calculated as following Equation 9.


(9) $$Y_{c}=E_{\mathrm{seq}}(v_{b})=\frac{1}{L\times M}\sum_{x=1}^{L}\sum_{y=1}^{M}v_{b}(x,y)$$

Where $v_{b}$ represent the input, $Y_{c}$ represent the data after squeeze operation, $E_{\mathrm{seq}}$ represent the squeeze function, L and M _denote the width and height of the feature map._ For Excitation Layer, here consider $V_{1}\in Q^{\frac{B}{s}\mathrm{yB}}$ and $V_{2}\in Q^{\mathrm{By}\frac{B}{s}}$ is the Weights of the first and second fully correlated layer, δ ReLU activation function. Excitation Layer are define as following Equation 10:


(10) $$P=\sigma (V_{2}\delta (V_{1}y))$$

Convolution is coupled with a residual connection, which retains valuable information from previous layers and helps reduce possible information loss during processing. It can be address vanishing and exploding gradients problems. It can calculated as following Equations 11, 12.


(11) $$z_{x}=E(j_{x},K_{x})+l(j_{x})$$

(12) $$y_{x+1}=e(v_{x})$$

Where E(•) represent the residual function, $y_{x}$ and $y_{x+1}$ represent the I/o of the residual unit, l(•) represent the identity mapping function. With the suggested method, the picture has been divided well.

### 2.4 Feature extraction using ensemble VGG assisted Mobile Net model

**Figure 3:**
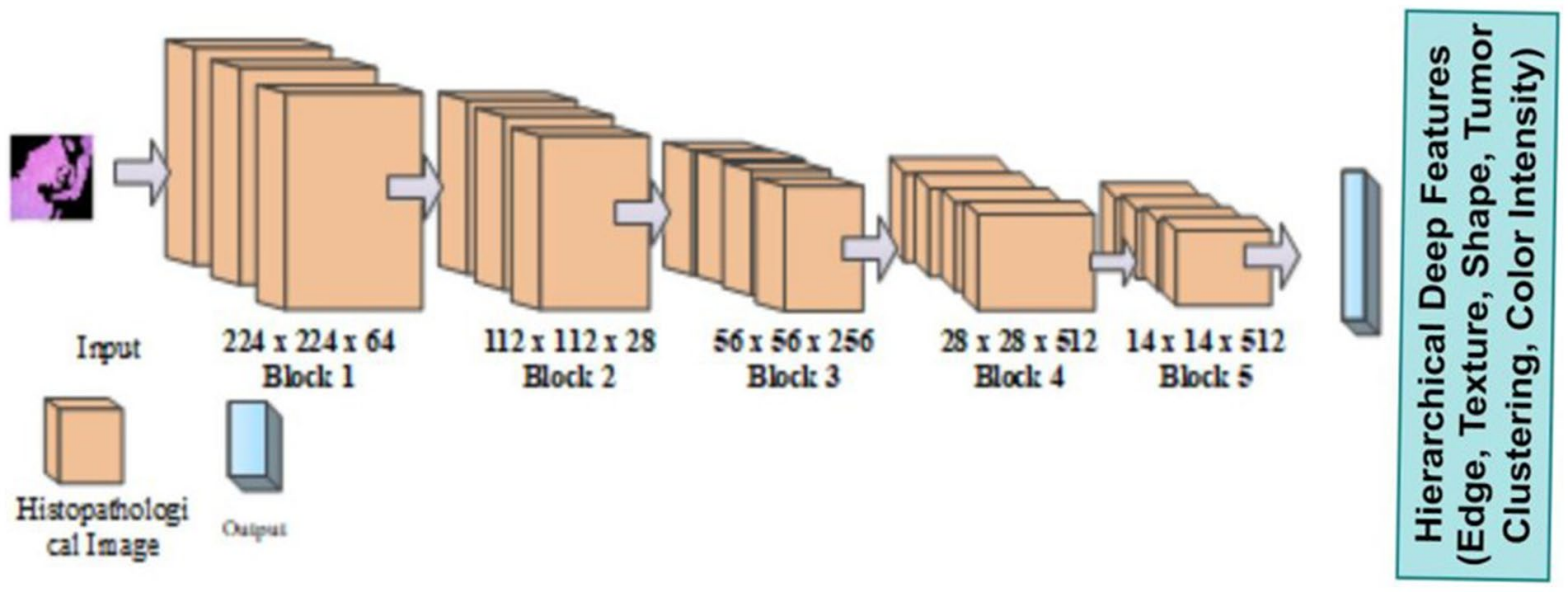
Architecture of VGG.

The Mobile VGG framework is built using deep separable curves. Existing methods such as ResNet, InceptionNet, and so on had some limitations, such as complex textural features, size, and shape for feature extraction. To overcome that limitation, introduce a novel VGG-Mob. The VGG (Albalawi et al., 2024) network was built with very small convolutional filters for image recognition. VGG 16 structure follows convolution and pooling layer. It contains Hierarchical Deep Features like Edge, Texture, Shape, Tumor Clustering, Color Intensity shown in Figure 3.

**Figure 4:**
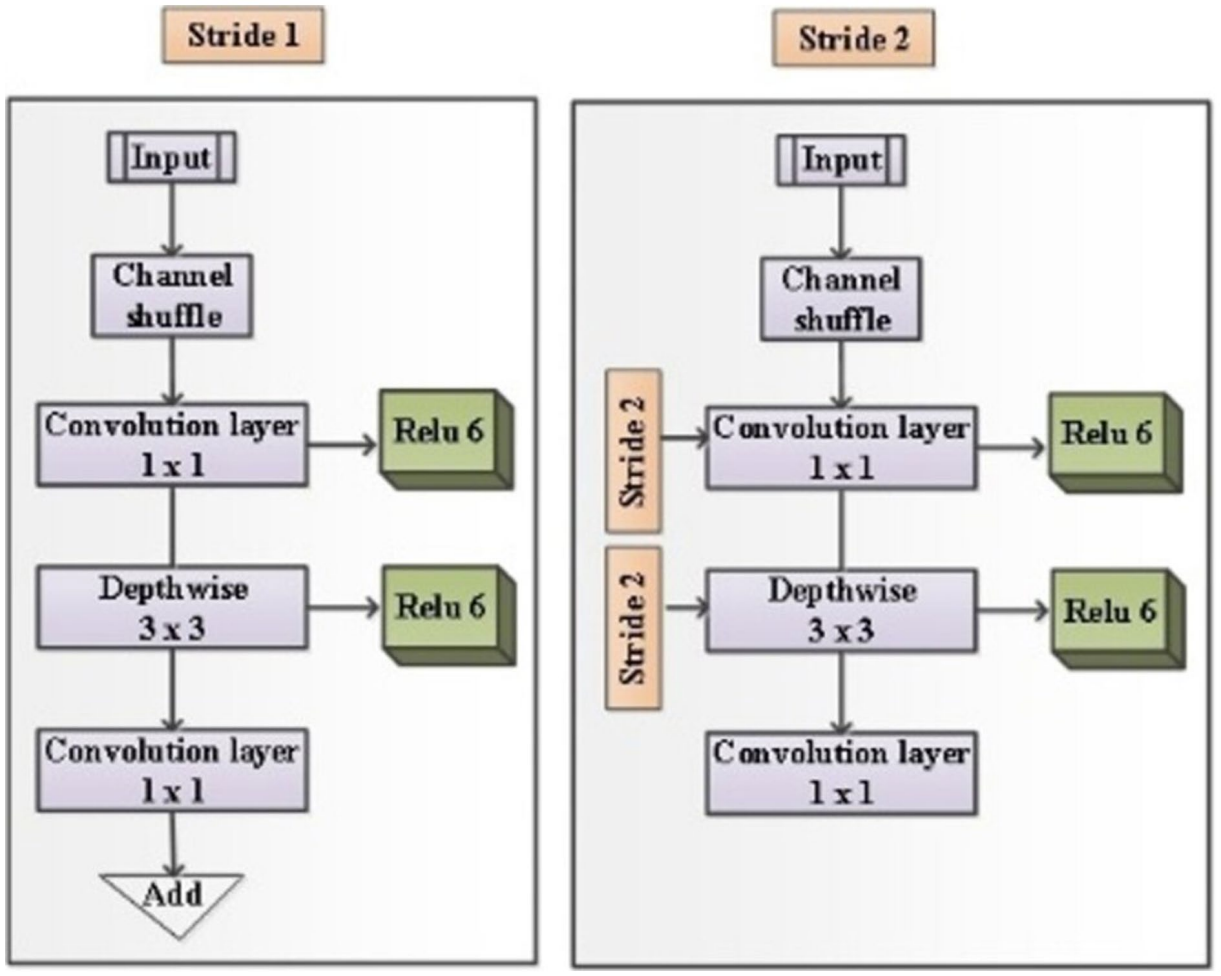
Architecture of Mobile Net.

It is built with three fully connected layers. The first and second layers are ReLU, and the third is Soft max-activated. Images can be obtained with an input layer of 224 × 224 pixels and this format has 16 layers and 138 Millions of parameters. MobileNetV2’s Ochoa-Ornelas et al. (2025) efficient architecture improves the model’s capacity to capture feature inter-class differences, making it highly suitable for clinical image analysis that relies on common visual features. Figure 4 shows the architecture of Mobile Net.

That has included several other pivotal architectural innovations like Depthwise Separable Convolutions, Inverted Residuals and Linear Bottlenecks, and ReLU6 Activation Function. In Depthwise Separable Convolutions, the operation separates the common basic convolution into two layers like depthwise convolution and point-wise convolution, which convolve the input channels individually and sum these filtered output images to create new level features. It can be represented as the following Equations 13, 14.


(13) $$x_{m,n,o}=\sum_{j=1}^{J}\sum_{k=1}^{K}y_{m+k-1,n+k-1,o}\cdot v_{j,k,o}$$

(14) $$y_{m,n}=\sum_{o=1}^{O}x_{m,n,o}*q_{o}$$

Where $v_{j,k,o}$ represent the depthwise convolution kernel, $x_{m,n,o}$ and $y_{m,n,o}$ represent “the input and output feature map,” J and K represent the dimensions of the kernel. Then $q_{o}$ weight parameter and $y_{m,n}$ shows the final output after point-wise convolution. MobileNetV2 uses inverse residuals in I/P thin moderate layers and extended intermediate of layers. Linear bottlenecks prevent nonlinearities such as ReLU information loss. Which can be defined as the following Equation 15.


(15) $$x=\max(0,V_{\exp}\cdot y)\cdot V_{\mathrm{pro}}$$

Where $V_{\mathrm{pro}}$ represent the projection matrix, $V_{\exp}$ represent the matrix of expansion. MobileNetV2 uses the ReLU6 activation function to reduce the size error and prevent information loss in low-PRE calculation, which is defined as the following Equation 16.


(16) ReLU6(y)=min(max(0,y))

Where y input tensor. Using the VGG-Mob model, the suggested method was able to successfully extract the feature.

### 2.5 Classification using deep optimized transformer encoder assisted dilated convolutional with global attention

**Figure 5:**
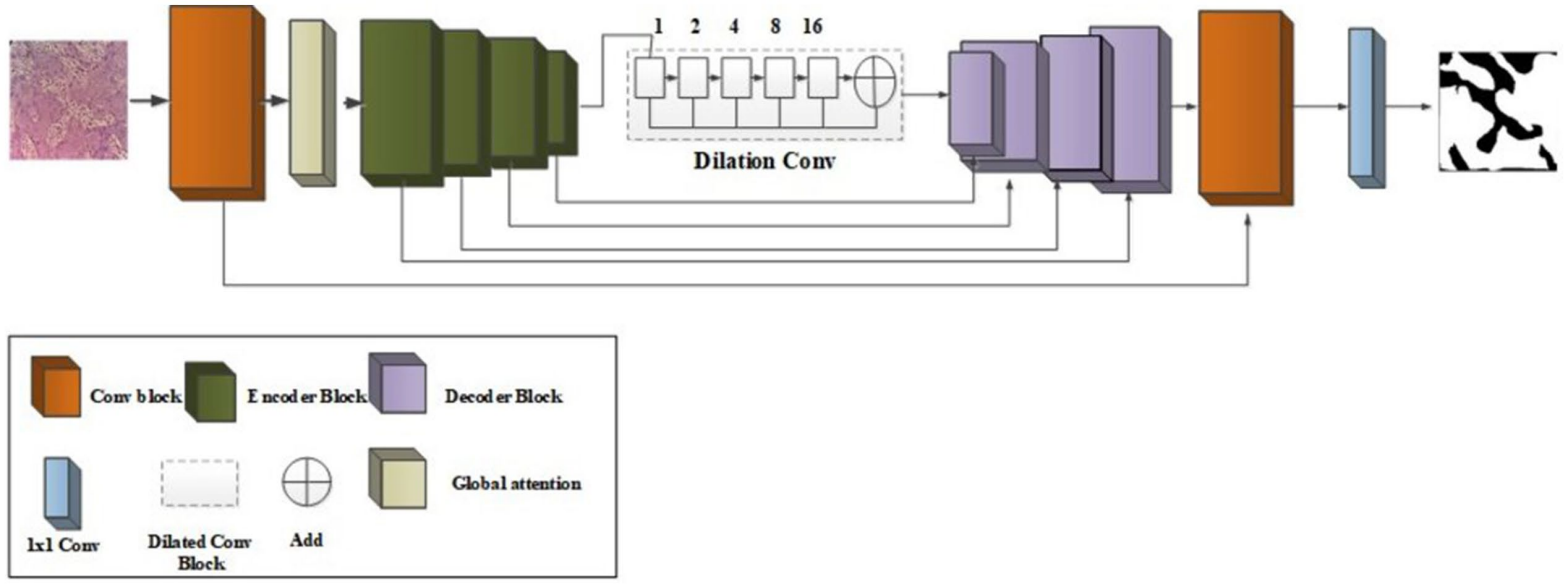
Architecture of DeTr-DiGAtt.

Several methods have been developed for OSCC grade classes, but the existing methods have some limitations, such as overfitting, class imbalance problems, and overtime duration. To overcome those problems, introduce a Novel DeTr-DiGAtt. Figure 5 shows the architecture of the deep novel DeTr-DiGAtt.

The input $Y\in {ℜ}^{M\times L}$ to the transformer encoder (Gao et al., 2022) consists of two modules namely multi-head self-focusing (MSA) and MLP modules, which is calculated according to the following Equations 17, 18.


(17) $$y_{m}^{\prime }=\mathrm{MSA}(\mathrm{LN}(y_{m-1}))+y_{m-1}$$

(18) $$y_{m}=\mathrm{MSA}(\mathrm{LN}(y_{m}))+y_{m}^{\prime },m=1,2,.\ldots M$$

Where LN _and_ MLP, Two layers utilize the GELU activation function for non-linearity, while Layer Normalization (LN) is applied to each sample. It is computed using the following Equation 19:


(19) $$\mathrm{LN}(y)=\frac{y-\mu }{\delta }\mathrm{p\gamma}+\beta$$

Where μ represent the mean derivation of feature, y represent the sample, δ represent the standard derivation of feature, p represent the element-wise dot operation, γ and β represent the learnable parameters of affine transformation. A dilated convolution layer is introduced to extract the high-level and fine low-level semantic information features. Dilated convolution layers have been shown to be a good alternative to segmentation tasks and pooling layers with significant improvement in ACC. In the expanded CNN, they use a pooling layer to control over-fitting and maintain in-variance, which is help reduces the spatial resolution information. It is Equation 20 given as following:


(20) $$x(j,k)=\sum_{n=1}^{J}\sum_{o=1}^{K}y(j+m\times n,k+m\times o)v(n,o)$$

Where v(n,o) represent the filter with J length and K width, x(j,k) represent the output of dilated convolution y(j,k) represent the input of dilated convolution and m represent the parameter of dilated rate. The traditional convolutional get 3×3 kernel size receptive field. Then two dilated convolutions get 5×5 and 7×7 kernel size receptive field. It can shows expand the respective field without loss of feature resolution. Global attention is a mechanism that calculates the attention weight of all elements of the input sequence. It was used to global context and capture long-range dependencies to gather the complex structure of images. It can be defined as following Equations 21, 22:


(21) $$G_{2}=P_{B}(G_{1})⊗G_{1}$$

(22) $$G_{3}=P_{q}(G_{2})⊗G_{21}$$

Where $G_{1},G_{2},P_{B},P_{q}\in {ℜ}^{L\times M\times N}$ , $P_{B}(G_{1})$ _map the input feature to get the channel weight feature map_ $P_{B}$ , $\left(G_{2}\right)$ represent the intermediate map _feature_, $P_{q}(G_{2})$ represent the spatial weight feature map multiplied with $\left(G_{2}\right)$ and $G_{3}$ represent the output feature map. Then ⊗ represent the multiplication between pixels. Then L , M and N represent the height, width and number of channels of the feature map. Finally, the classifier model has been carefully tuned for hyperparameters; however, the current optimization techniques, like Grey Wolf Optimizer, Particle Swarm Optimization, and Satin Bowerbird Optimizer, have certain drawbacks, e.g., complex structures, decreased effectiveness, vulnerability to capture, and higher computational complexity. In order to overcome such concern, suggest a novel Ad-GreLop. Greylag Goose Optimization (GGO) approach (Elshewey et al., 2025) is a meta-heuristic optimization method that mimics the feeding pattern of Greylag geese and adjusts their locations to determine the optimal solution. Exploration stage in the GGO approach gives top priority to determining promising areas in the search space while avoiding stagnation both in location and inner goals by progressing towards an optimum solution. The GGO re-evaluates agent placements based on specific calculations and maximizes these locations as depicted below Equation 23.


(23) $$Y(n+1)=Y^{*}(n)-B\cdot |D\cdot Y^{*}(n)-Y(n)|$$

Where B and C represent the updating vector, Y(n) represents the agent’s position at $n^{\mathrm{th}}$ , $Y^{*}(n)$ represent the leader’s position. Then $N_{\text{paddle}1},N_{\text{paddle}2}$ and $N_{\text{paddle}3}$ represents the three randomly chosen search agents, which is used to upgrade agent locations. It can be defined as the following Equations 24, 25.


(24) $$\begin{array}{l} Y(n+1)=v_{1}\cdot Y_{\text{paddle}1}+x\cdot v_{1}\cdot (Y_{\text{paddle}2}-Y_{\text{paddle}3})\\ +(1-x).v_{3}(Y-Y_{\text{paddle}1}) \end{array}$$

(25) z=1−(t/rmax)2

Where $v_{1},v_{2}$ and $v_{3}$ represents the upgraded in the range of [0, 2]. Then rma represents the number of maximum iterations, t represents the iteration number. Progressing toward the best optimal solution is define as the following Equations 26–28.


(26) $$Y_{1}=Y_{\mathrm{sen}1}-D_{1}.|E_{1}.Y_{\mathrm{sen}1}-Y|$$

(27) $$Y_{2}=Y_{\mathrm{sen}2}-D_{2}.|E_{2}.Y_{\mathrm{sen}2}-Y|$$

(28) $$Y_{3}=Y_{\mathrm{sen}3}-D_{3}.|E_{3}.Y_{\mathrm{sen}3}-Y|$$

Where $D_{1}$ , $D_{2}$ , $D_{3}$ $E_{1}$ , $E_{2}$ and $E_{3}$ represents the updates vector and $Y_{\mathrm{sen}1}$ , $Y_{\mathrm{sen}2}$ and $Y_{\mathrm{sen}3}$ represent the anticipated position of the prey based on three sentry solutions. GGO periodically rotates leadership among individuals in the population to prevent stagnation. It can be define as following Equation 29.


(29) $$X_{\text{leader}}^{n+1}=X_{\text{leader}}^{n+1}+m.(X_{\text{leader}}^{n}-X_{\text{leader}}^{n})$$

Where $Y_{t}^{n}$ represents the $t^{\mathrm{th}}$ goose position in the population at $n^{\mathrm{th}}$ iteration, $X_{\text{leader}}^{n+1}$ represent the leader position and $X_{\text{leader}}^{n+1}$ represent the randomly chosen position from the population and m represent the random factor in [0, 1]. Then chaotic function has been add to update the grey lag goose optimization algorithm ch⋅ represent the chaotic function in the range [0, 1]. It can be define as the following Equations 30, 31:


(30) $$X_{\text{leader}}^{n+1}=X_{\text{leader}}^{n+1}+\mathrm{ch}\cdot (X_{\text{leader}}^{n}-X_{\text{leader}}^{n})$$

(31) $$\mathrm{ch}=P_{s+1}=\cos(s\cos^{-1}(P_{q}))$$

Where s represent the chaotic index sequence and $P_{q}$ represent the number of $q^{\mathrm{th}}$ element. In fitness function, the model’s ability to identify entire negative or positive case instances, which is calculated as the following Equation 32.


(32) $$\mathrm{Acc}=\frac{\mathrm{UR}+\mathrm{UV}}{\mathrm{UR}+\mathrm{UV}+\mathrm{GR}+\mathrm{GV}}$$

Where UR represent the true positive, UV represents the true negative, GR represent the false positive and GV represent the false negative. Algorithm 1 represents the Pseudocode for Ad-GreLop.

**ALGORITHM 1:**
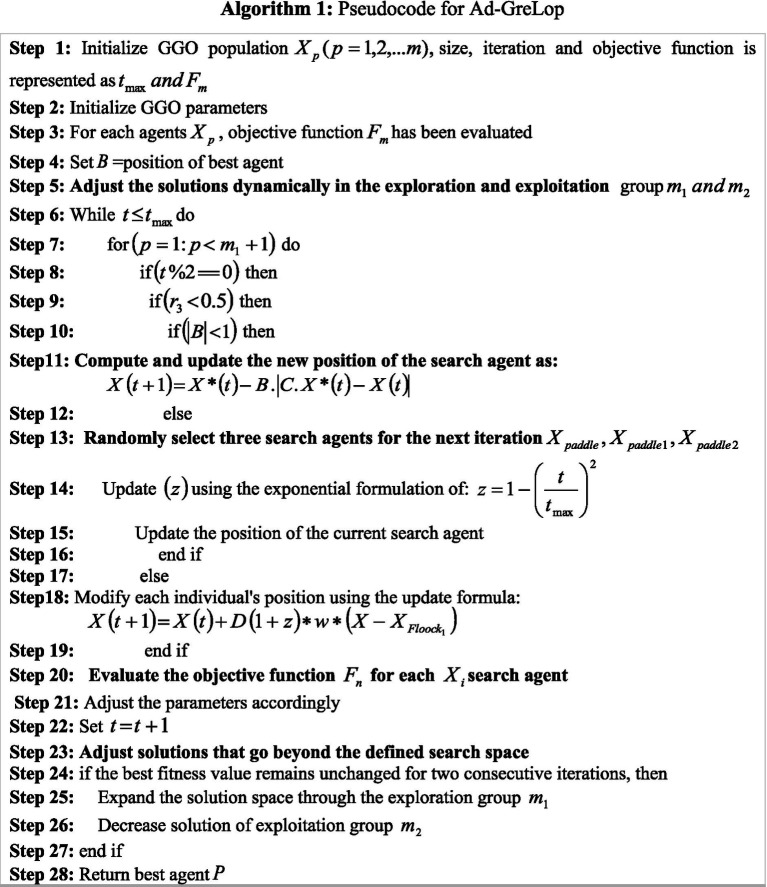
Pseudocode for Ad-GreLop.

The suggested technique has been successfully categorised with DeTr-DiGAtt and the Adaptive Grey Lag Goose Optimization Algorithm.

## 3 Result and discussion

The proposed methodology is compared with other existing methodologies are “ResNet, Inception Net, Xception Net, AlexNet, U-Net, V-Net, SegNet along with VGG16 and ResNet. The proposed method has been evaluated using its dataset. Afterwards, some performance evaluation measures like ACC, PRE, recall, F1 score, specificity, and dice score were utilized for OSCC classification (Table 1).

**Table 1:** Existing methods analysis.

Reference	Method	Limitation
Wako et al. (2022)	CNN based TL model	Absence of hybrid model leads to reduce the model performances.
Albalawi et al. (2024)	EfficientNet B3	Broader dataset was not used in this research was one of the major limitations in this model.
Peng et al. (2024)	DL	Very limited amount of ACC were obtained for this approach was one of the major limitations.
Das et al. (2024)	CNN	Binary classification has performed, there was an absence of multi-class approach in this model.
Flügge et al. (2023)	DL based approach based on Swin-Transformer (ST)	This method had lack of real time environment problem.
Li et al. (2024)	DL	The suggested method had insufficient data collection.

### 3.1 Dataset description

The dataset was collected from vishnu dental college Bhimavaram, AP, contains 34 moderately differentiated samples, 11 poorly differentiated samples, and 35 well-differentiated sample images. Before augmentation, the dataset included 34 moderate, 11 poor, and 34 well-differentiated sample images. After augmentation, the dataset was expanded to 204 moderate, 66 poor, and 204 well-differentiated sample images. In Table 2 shows all grading images of segmented and processed. The efficacy of hyperparameter optimization for the suggested methodology encompasses the following primary parameters: a dropout rate of 0.5, ReLU activation function, global average pooling enabled, a learning rate of 0.001, a patch size of 32, and 300 training epochs. All these settings were optimized to enhance the ACC and efficiency of the OSCC classification model. Ethical clearance number for Real Time Dataset 8,758/IEC/2023.

**Table 2:** Sample of input, pre-processed and segmented images.

Sample	Original image	Pre-processed image	Segmented image
Moderate	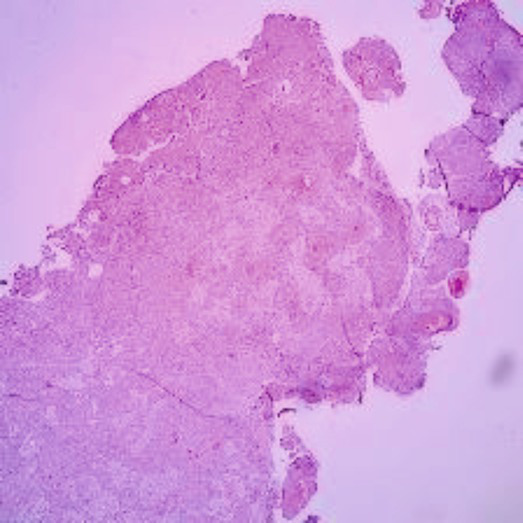	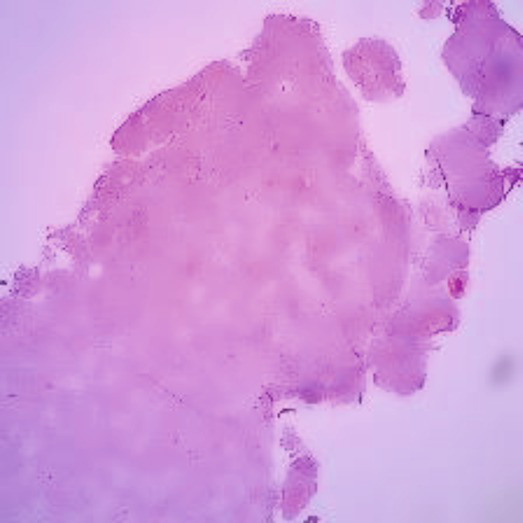	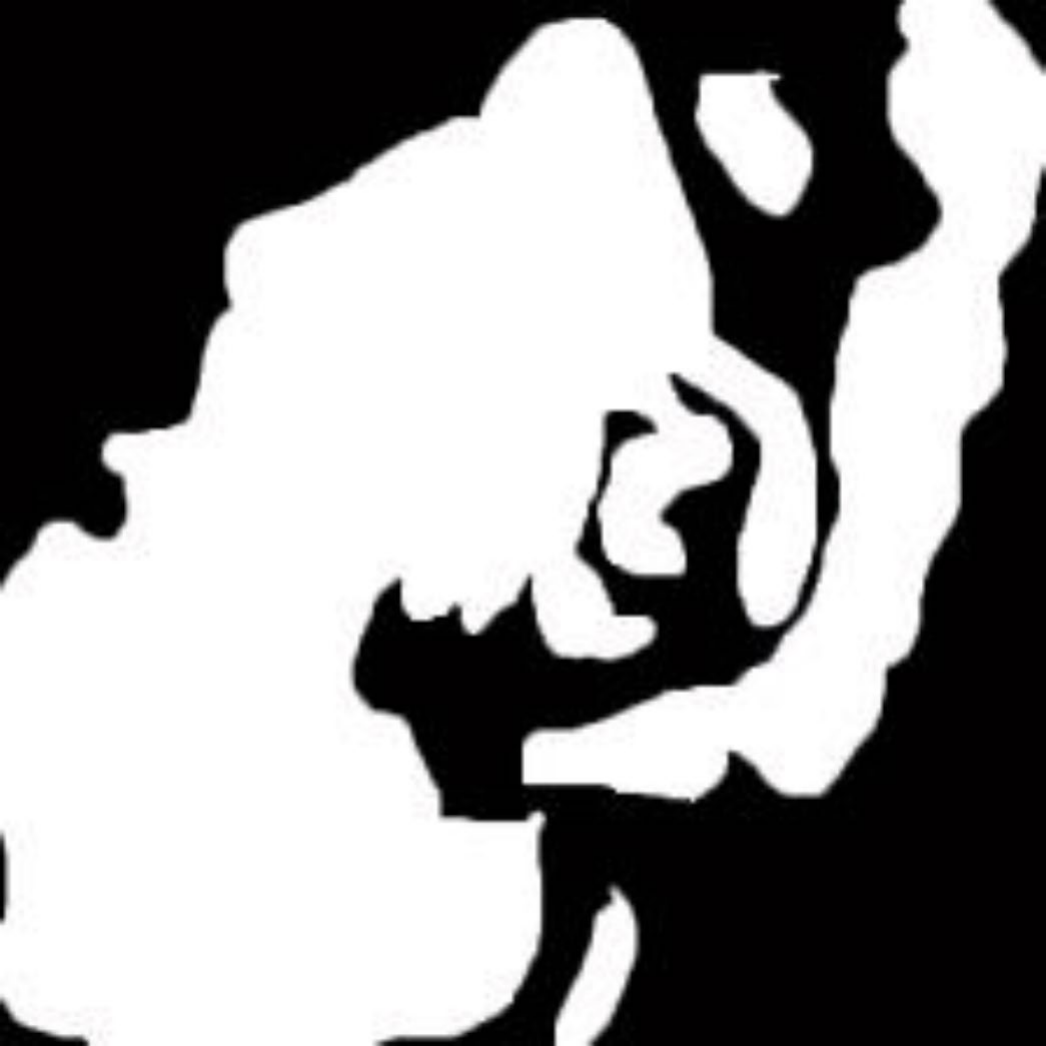
Poor	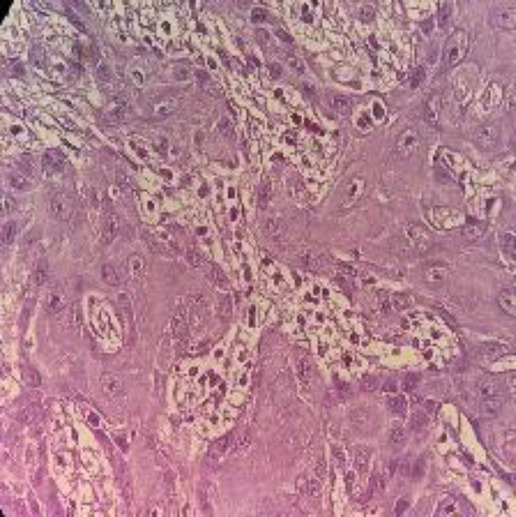	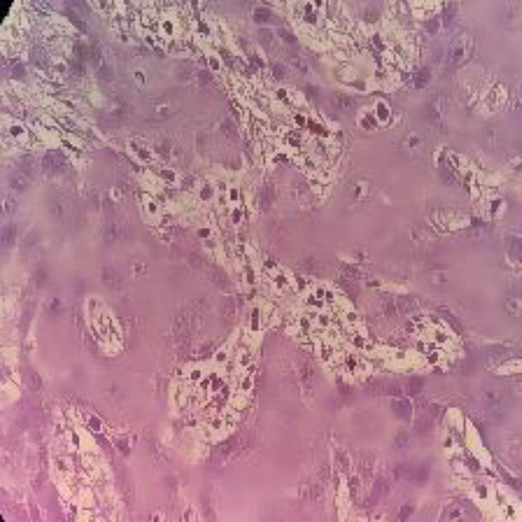	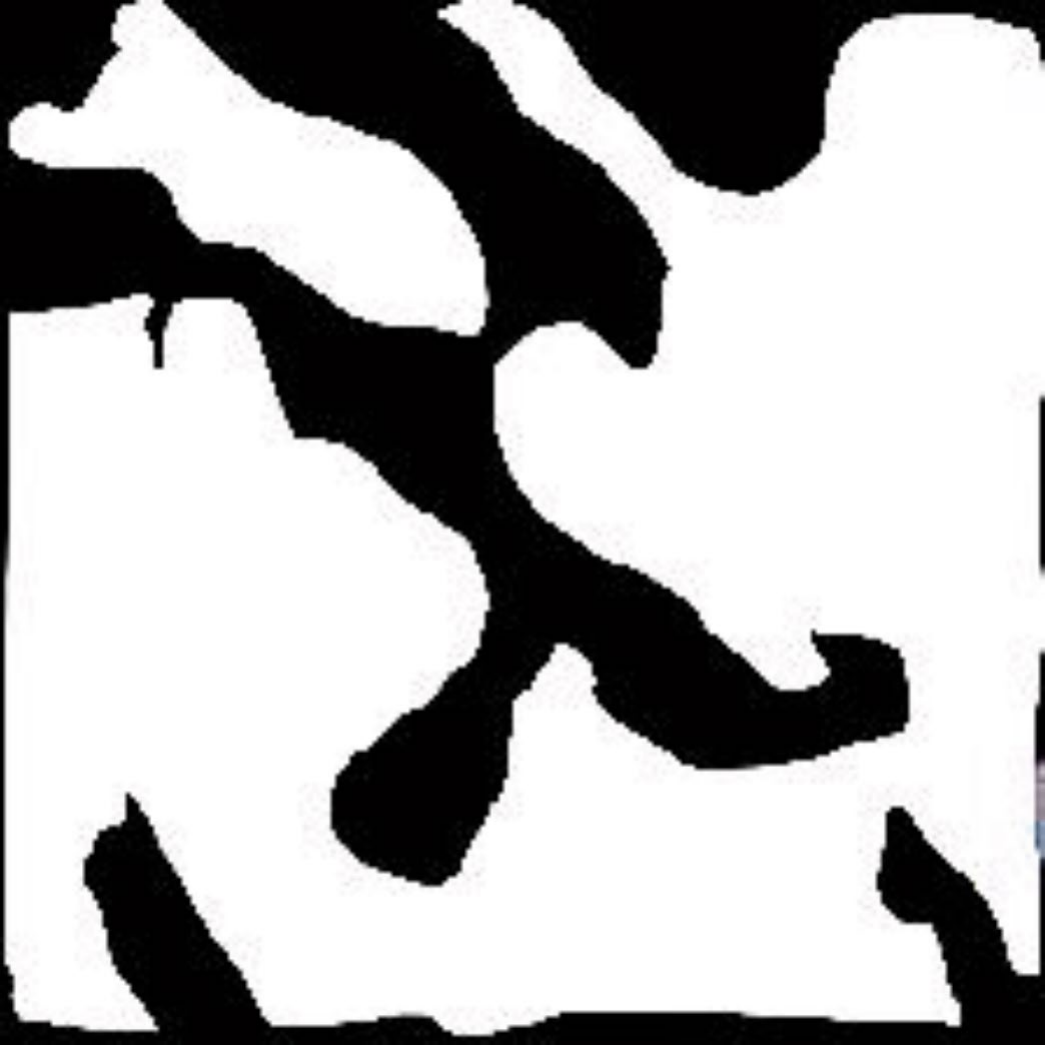
Well	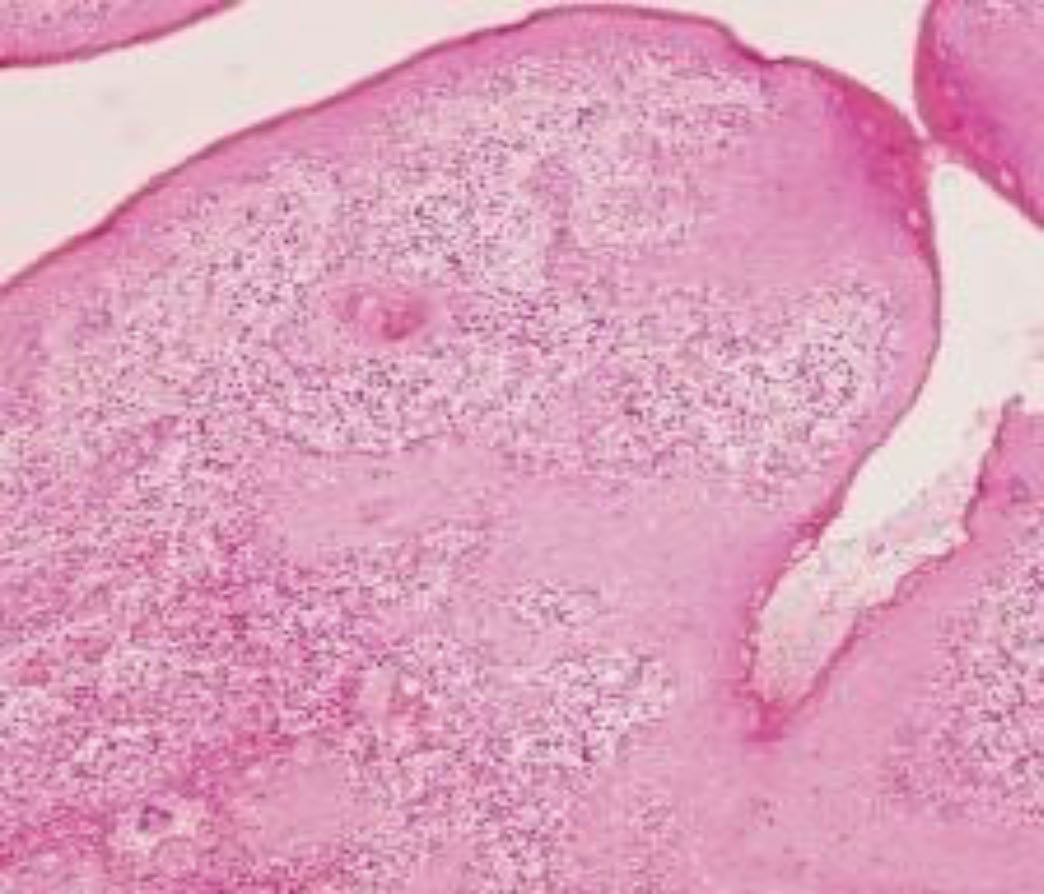	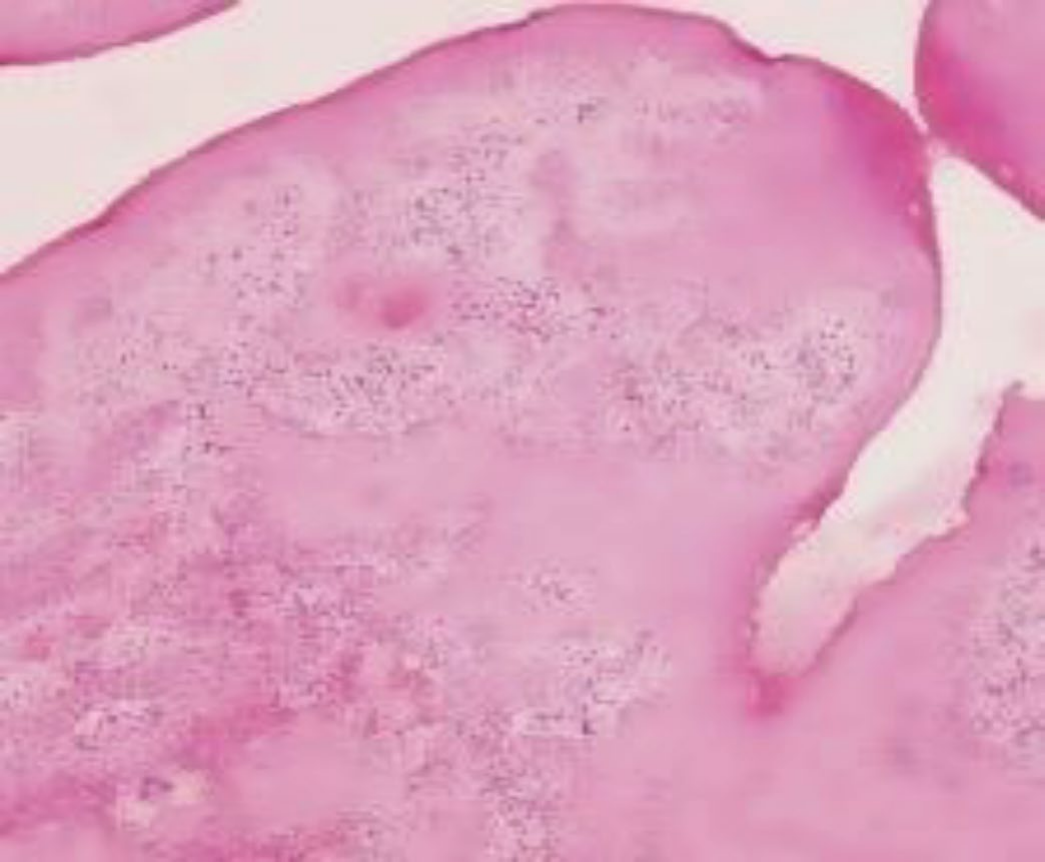	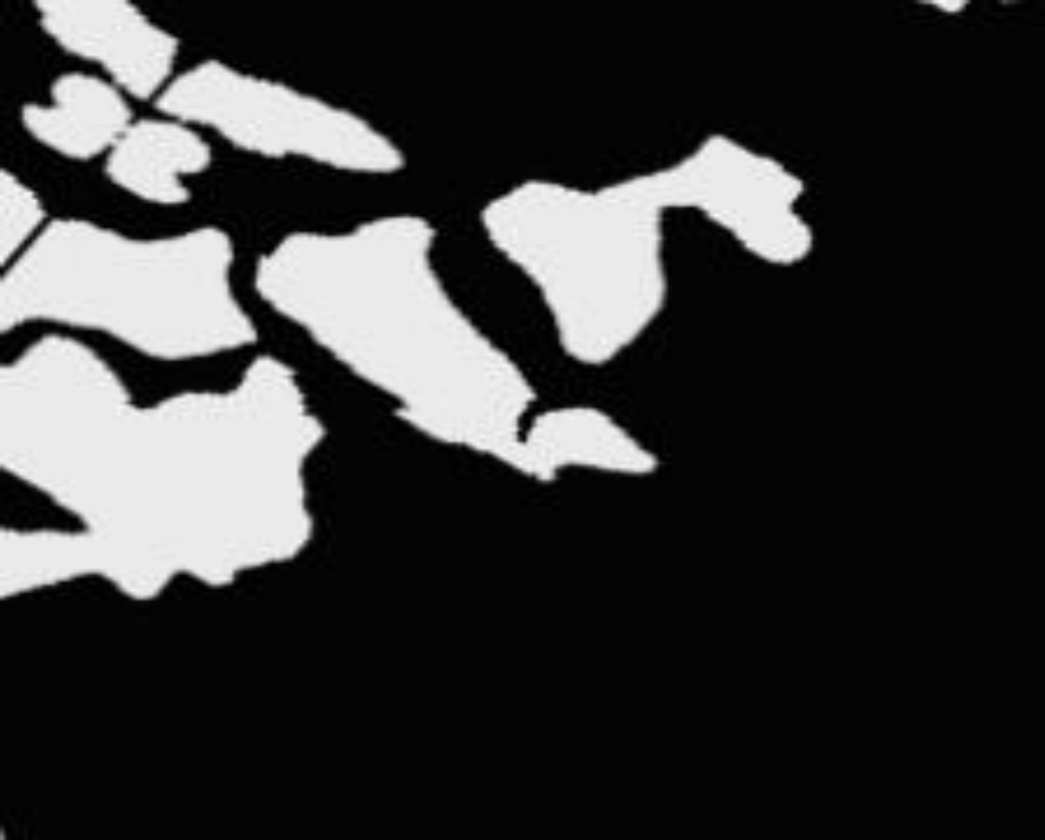

#### 3.1.1 Performance analysis of segmentation

**Figure 6:**
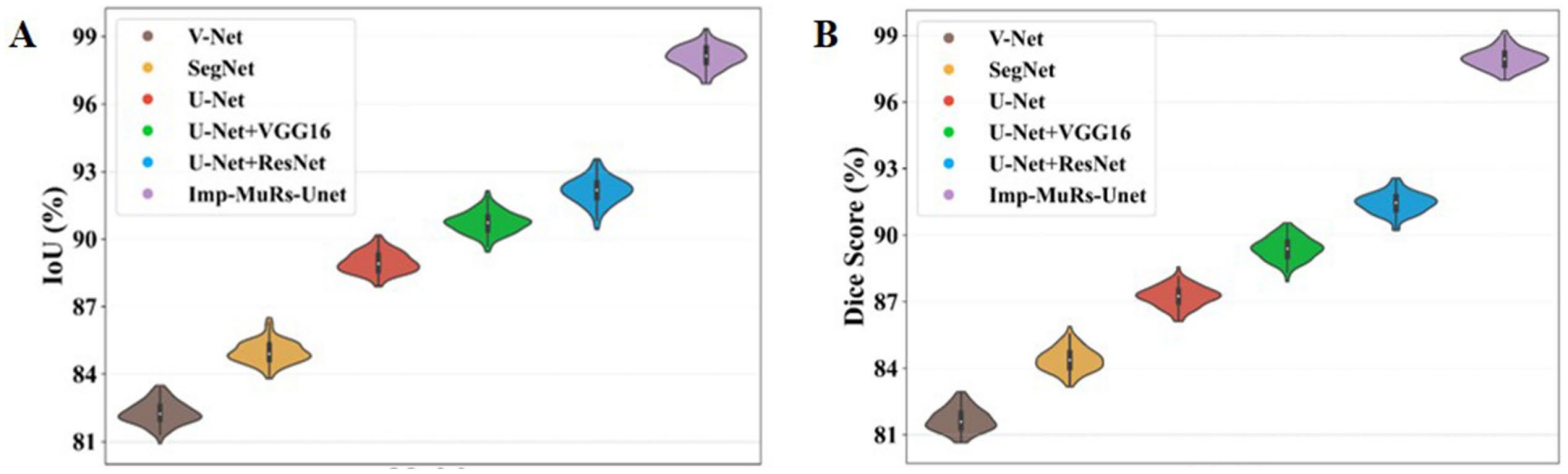
**(A,B)** Performance analysis of IoU and dice score.

Figure 6 illustrates the comparative performance of the proposed hybrid segmentation approach with existing methods. In Figure 6A, the proposed method achieves an IoU of 98.08%, outperforming U-Net + ResNet (92.20%) and VGG16 + U-Net (90.72%). This demonstrates the stronger generalization capability of the proposed model compared to prior approaches, which often struggled with segmentation quality. Similarly, Figure 6B compares Dice coefficients, where the proposed method attains a Dice score of 97.97%, significantly higher than U-Net (87.24%) and SegNet (84.41%). These results highlight the ability of the proposed model to overcome limitations of earlier methods, such as low-quality segmentation and reduced accuracy.

**Figure 7:**
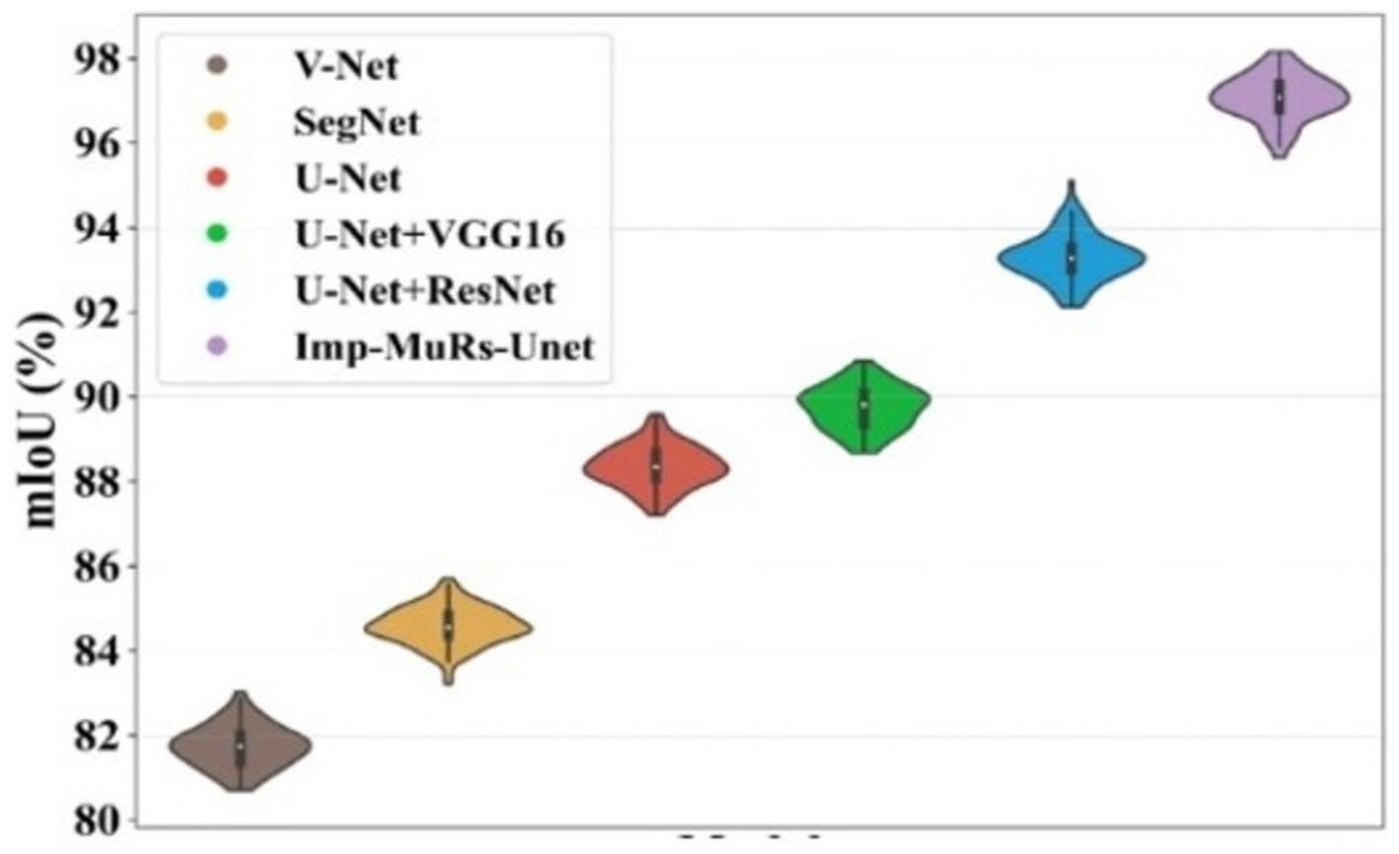
Performance analysis of mIoU.

Figure 7 presents the mean Intersection-over-Union (mIoU) analysis. The proposed approach achieves an mIoU exceeding 97.01%, while V-Net and U-Net record comparatively lower values of 81.69% and 88.39%, respectively. This indicates that existing segmentation models often face challenges such as increased complexity, limited applicability, and dependency on large annotated datasets. In contrast, the proposed method consistently delivers superior segmentation performance across different evaluation metrics. A consolidated comparison of these results is further provided in Table 3, emphasizing the robustness of the proposed approach.

**Table 3:** Segmentation analysis for proposed method.

Methods	IoU (%)	Dice score (%)	mIoU (%)
Imp-MuRs-Unet	98.08	97.97	97.01
U-Net + ResNet	92.20	91.49	93.28
U-Net + VGG16	90.72	89.34	89.75
U-Net	89	87.24	88.39
SegNet	85	84.41	84.64
V-Net	82.26	81.69	81.69

#### 3.1.2 Performance analysis for classification

**Figure 8:**
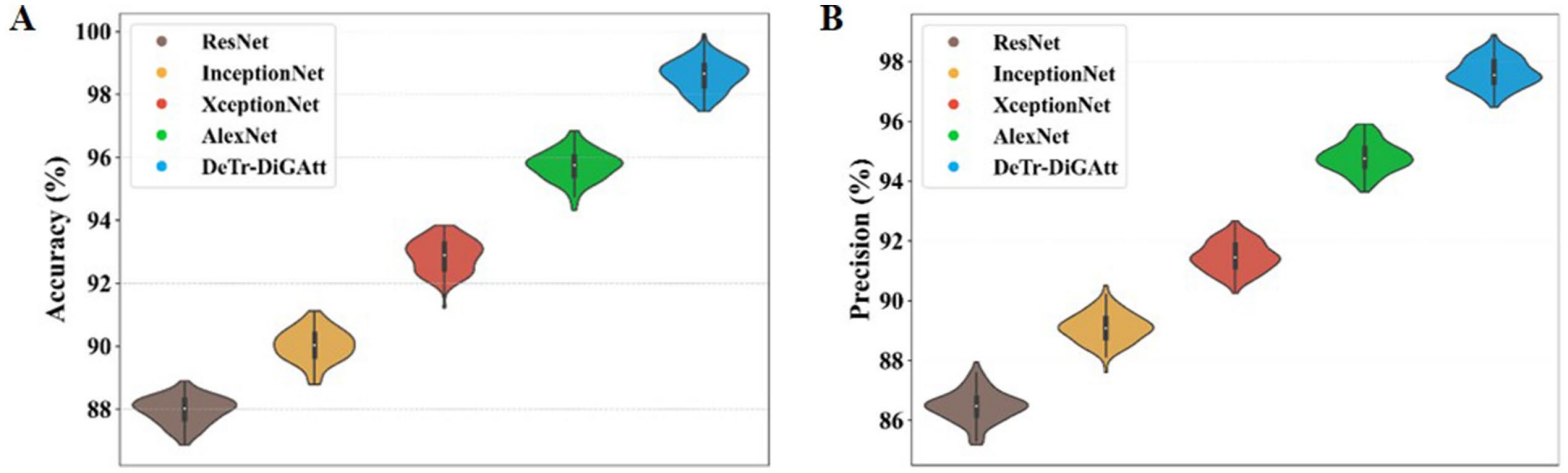
**(A,B)** Performance analysis of ACC and precision.

This section provides a comparison of the suggested methodology with several contemporary techniques. Figure 8 illustrates the performance of ACC and PRE.

Figure 8 presents the classification performance of the proposed method compared to existing models. In Figure 8A, the proposed strategy achieves an accuracy (ACC) of 98.59%, outperforming AlexNet (95.74%) and XceptionNet (92.91%). This confirms the superior classification ability of the proposed method, while earlier techniques such as AlexNet also exhibited longer computational times. Figure 8B illustrates the precision (PRE) values, where the proposed approach attains 97.53%, significantly higher than XceptionNet (91.35%) and InceptionNet (89.13%). The lower accuracy and precision of InceptionNet highlight its increased complexity and reduced effectiveness in OSCC classification.

**Figure 9:**
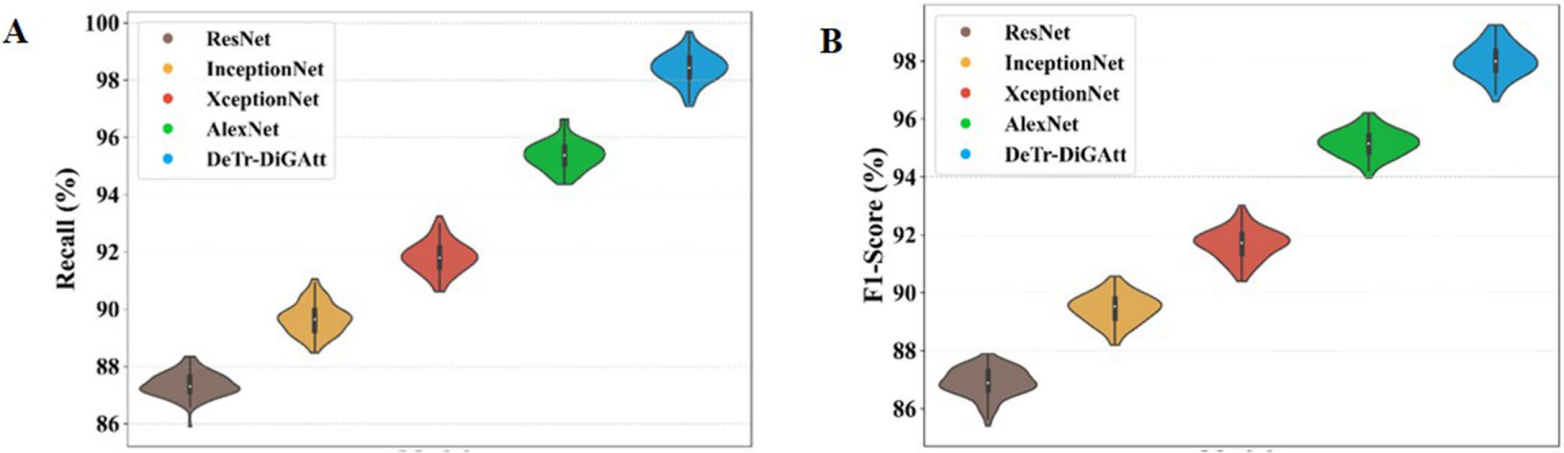
**(A,B)** Performance of recall and F1- score for proposed method.

Figure 9 evaluates additional performance metrics of Recall and F1-score. As shown in Figure 9A, the proposed method achieves a recall value of 98.45%, whereas AlexNet and InceptionNet obtain only 89.76% and 95.35%, respectively. This improvement demonstrates the ability of the proposed approach to handle class imbalance issues that hindered prior models. Figure 9B shows F1-score comparisons, where the proposed method records 97.99%, clearly surpassing InceptionNet (89.44%) and ResNet (86.98%). The higher F1-score indicates the robustness of the proposed model in minimizing both false positives and false negatives, thereby improving the detection of OSCC cases.

**Figure 10:**
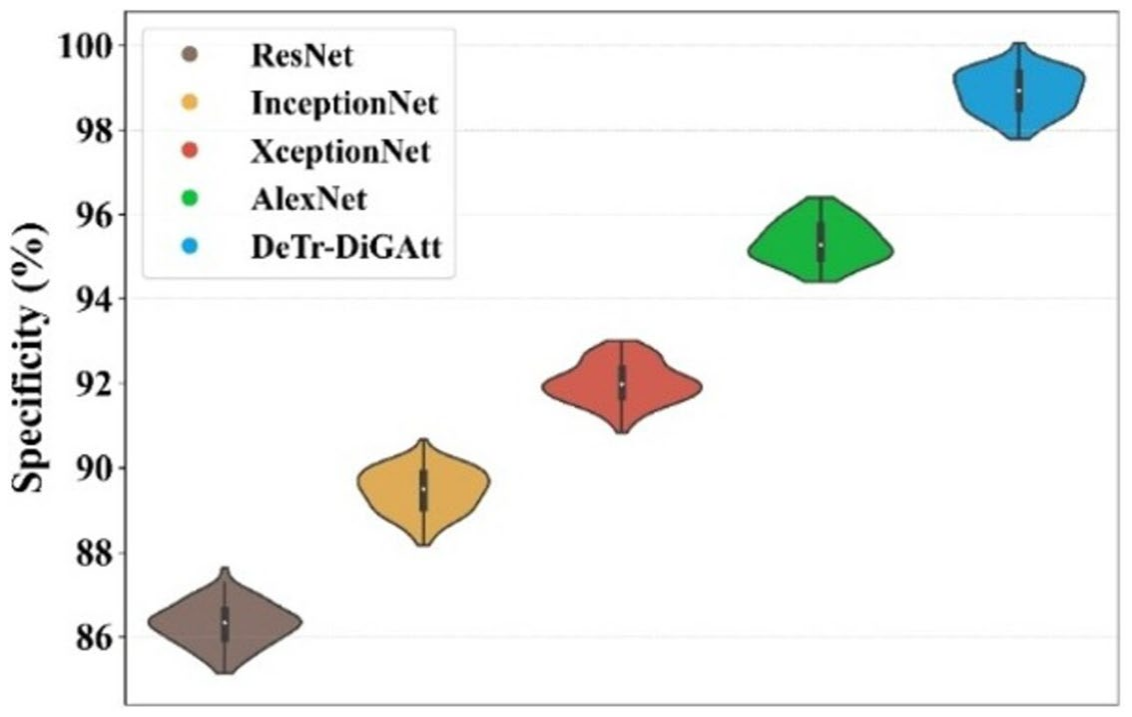
Performance analysis of specificity.

**Figure 11:**
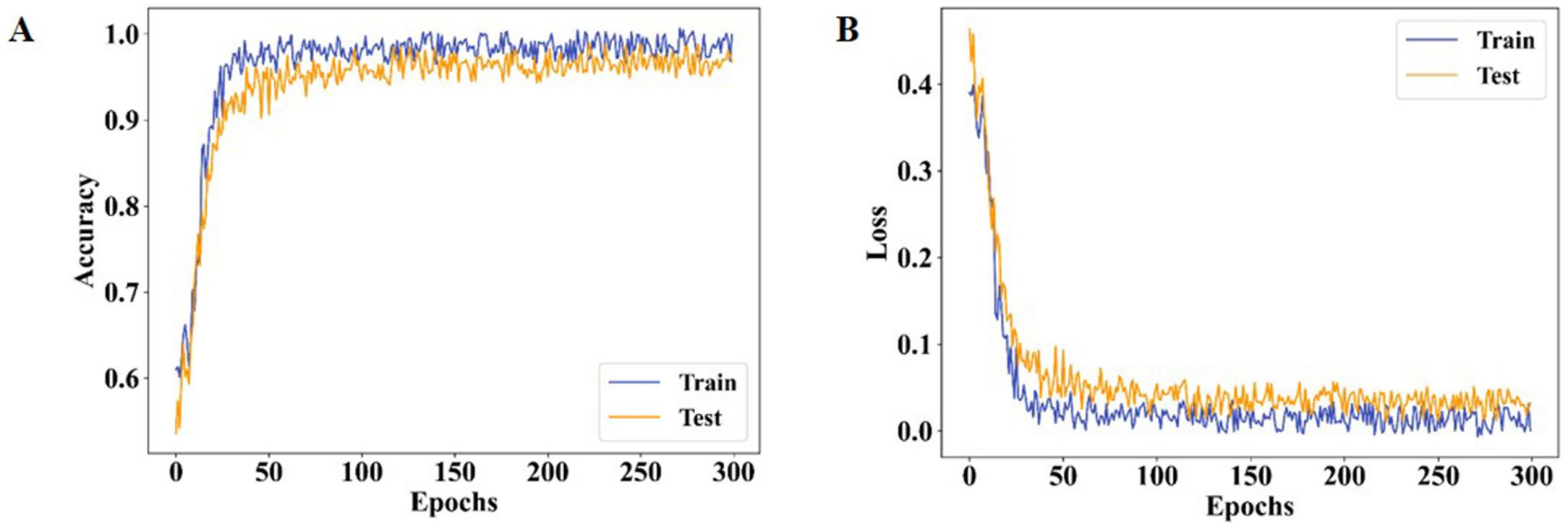
Performance analysis of both training and testing **(A)** ACC and **(B)** loss for proposed method.

Figure 10 highlights the specificity analysis. The proposed method achieves a specificity of 98.96%, which is higher than AlexNet (95.41%) and XceptionNet (92.07%). This demonstrates the enhanced ability of the proposed approach to correctly identify negative cases, reducing misclassification rates and providing more reliable predictions. Prior models often suffered from class imbalance, reducing their specificity and overall classification stability. Figure 11 presents the training and testing performance across epochs. In Figure 11A, the proposed model achieves near-perfect accuracy for both training and testing after approximately 300 epochs, reflecting its strong generalization ability. Figure 11B shows that both training and testing loss values remain below 1 within the same epoch range, confirming the stability and efficiency of the learning process.

**Figure 12:**
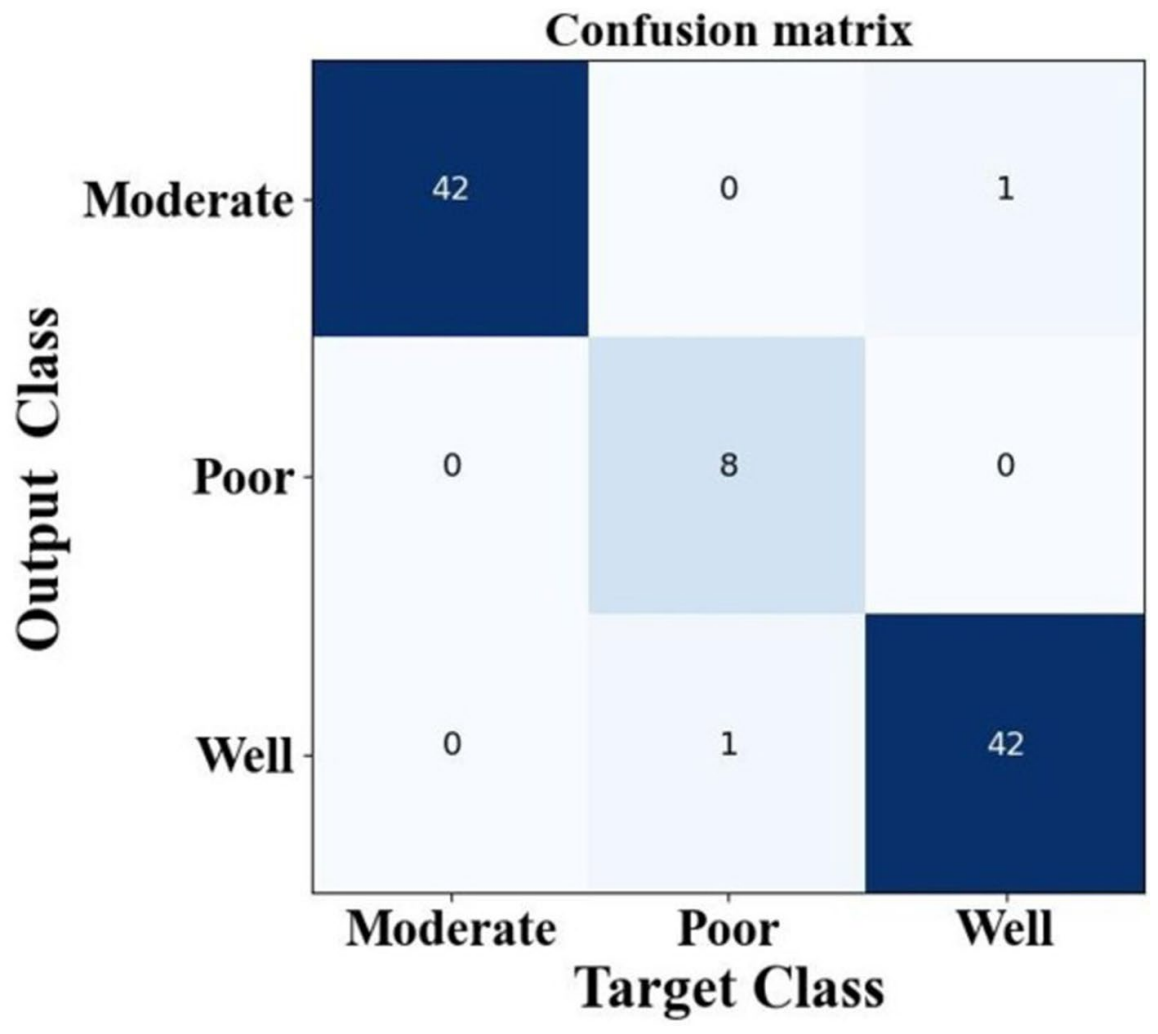
Performance analysis of confusion matrix of proposed method.

Figure 12 provides the confusion matrix for the three-class classification task. The distribution clearly indicates that the proposed method minimizes misclassifications across all OSCC grades, supporting its robustness and practical applicability for accurate disease classification.

To ensure that the observed performance improvements are not due to chance, the proposed model was evaluated using 5-fold cross-validation. Additionally, a paired *t*-test was conducted between the proposed model and baseline methods. The improvements in IoU, ACC, F1-score, and specificity were found to be statistically significant (*p* < 0.05). This confirms that the high scores achieved by the proposed method represent true performance gains rather than random variations or dataset bias.

To identified the target classes such as well, moderate, and poor, which help to improve the OSCC Grading Classification. The method has been obtaining total target class samples of moderate at 42 samples, poor at eight samples, and well at 42 samples. The proposed method obtains a moderate target class of 41 samples, a poor target class obtains eight samples and well obtains 41 samples. The suggested design has been acquiring superior target classification samples, hence enhancing the performance of the present model. The suggested classification performance indicators provide superior results relative to other techniques. Table 4 presents the categorization performance results of the study.

**Table 4:** Performance classification of proposed method.

Model	Proposed model	AlexNet	XceptionNet	InceptionNet	ResNet
ACC (%)	98.59	95.74	92.91	90.07	87.94
Precision (%)	97.53	94.89	91.35	89.13	86.53
Recall (%)	98.45	95.35	91.86	89.76	87.44
F1-score (%)	97.99	95.12	91.60	89.44	86.98
Specificity (%)	98.96	95.41	92.07	89.45	86.29

Here a comprehensive discussion was provided on OSCC Grading Classification. The proposed compared with multiple methods for analyzing the better classification. Traditional methods have some limitations, such as handling large volumes of image data, lack of availability and generality during segmentation, poor contrast, and so on. To overcome that limitation, introduce a novel DeTr-DiGAtt for OSCC classification. The proposed method used the GAN model to control the overfitting problems. The imp-MuRs-Unet method was used to identify affected regions accurately for segmentation.

## 4 Conclusion

This study presents a unique DeTr-DiGAtt model for the categorisation of OSCC. The proposal involves collecting the input picture using its dataset. The GAN model was used for the data augmentation mechanism. The ad-BF method was used in the Pre-processing stage. After reprocessing, Imp-MuRs-Unet was used for segmentation. Then VGG-Mob model was used for feature extraction. The deTr-DiGAtt method was used for classification. Ad-GreLop was used to optimize for fine tune parameters in the classifier model. This available method has ACC, PRE, recall, dice score, and IoM of 98.59%, 97.53%, 98.45%, 97.97%, and 98.08%. In future work, explainable techniques will be added to improve the ACC of OSCC classification more effectively.

## Data Availability

The original contributions presented in the study are included in the article/supplementary material, further inquiries can be directed to the corresponding author.
